# Hughes’ Clinical Introduction to the Practice of Auscultation

**Published:** 1854-11

**Authors:** 


**Hughes' Clinical Introduction to the Practice of Auscultation.**

Who has not this little work on physical diagnosis? If there be one, let him supply the *deficit* at once. It is small, and so are valuable jewels generally, and this is one of the purest water. It is a *multum in parvo* offering, and should have a conspicuous place in a medical library, but never hidden by being "overlaid" by other and more cumbrous works. It is convenient for reference by the practitioner whose engagements will not afford him time to examine into works more elaborate; and to the student it is an invaluable text book which should be memorised almost. To understand well this work he will have laid a sure foundation for ultimate superiority in physical diagnosis. Published by Blanchard & Lea, Phila.

